# Identifying stably expressed housekeeping genes in the endometrium of fertile women, women with recurrent implantation failure and recurrent miscarriages

**DOI:** 10.1038/s41598-017-07901-6

**Published:** 2017-11-01

**Authors:** Linden Stocker, Felino Cagampang, Ying Cheong

**Affiliations:** 1 University Hospital Southampton NHS Foundation Trust, Princess Anne Hospital, Division of Women and Newborn, Coxford Road, Southampton, SO16 5YA UK; 2 Institute of Developmental Sciences, University of Southampton, Faculty of Medicine, Tremona Road, Southampton, SO16 6YD UK; 3 Complete Fertility Centre, Princess Anne Hospital, Division of Women and Newborn, Coxford Road, Southampton, SO16 5YA UK

## Abstract

Housekeeping genes (HKG) are presumed to be constitutively expressed throughout tissue types but recent studies have shown they vary with pathophysiology. Often, validation of appropriate HKG is not made. There is no consensus on which HKGs are most stably expressed in endometrial tissue so this study aimed to identify the most stable HKG in the endometrium of women with recurrent implantation failure (RIF) and recurrent miscarriages (RM). Inclusion criteria were women between 25–45 years (n = 45) suffering recurrent miscarriage (RM), recurrent implantation failure (RIF) or fertile controls. Endometrial biopsies were taken and total RNA extraction, cDNA synthesis and PCR was performed using 10 candidate HKG. The genes were arranged in terms of stability and normalisation was determined. Several HKGs not previously tested in endometrial samples were found to be more stable than those previously identified as the most stable. Of these, the 5 most stable HKG (in order of stability) were *Prdm4* (PR domain 4) > *Ube4a* (Ubiquitin-Conjugating Enzyme 4a) > *Enox2* (Ecto-NOX Disulfide-Thiol Exchanger 2) > *Ube2d2* (Ubiquitin-conjugating enzyme E2D 2) > *Actb* (Actin beta). We therefore recommend using at least four of the aforementioned HKG for normalisation of endometrial tissues taken from patients with RM and RIF.

## Introduction

The measurement of mRNA expression is a widely accepted and useful method of describing and quantifying gene expression in any tissue. It offers a high-turnover and the accurate expression profiling of selected genes. This offers a means of investigating tissue such as the endometrium to allow the identification of markers that could potentially play a role in endometrial function, implantation of an embryo and continuation of successful pregnancy.

The use of mRNA expression by quantitative reverse transcription polymerase chain reaction (qRT-PCR) requires normalisation to constitutively expressed genes, known as housekeeping genes (HKG). HKG are genes that are required for the maintenance of basal cellular functions that are essential for the existence of a cell, regardless of its specific role in the tissue or organism^1^. Comparison of the gene of interest to these conserved HKG is vital to adjust for potential experimental variables such as the quantity of starting material, enzymatic activity and any differences in overall transcription between tissues^2^. This is especially prudent in cells undergoing growth and differentiation, often driven by subtle changes in gene expression. Thus, it is ideal to use HKG that are sufficiently expressed in the tissue of interest, have minimal variability and high stability irrespective of physiological or pathological conditions.

The most commonly used HKG have been shown to vary considerably across samples and tissues^2,3^. This includes glyceraldehyde-3-phosphate dehydrogenase (*Gapdh*), but has now been demonstrated to be less stably expressed in some tissues than is generally assumed^4,5^. Despite this, validation of the presumed stability of HKG in tissues such as the endometrium is often not made because it renders normalisation to tissue type unnecessary^6,7^. A large part of the genome is expressed at a low basal level in all tissues^8^ and therefore when considering using a ubiquitous gene to be the defining feature of a HKG, one should either look for all genes that are expressed above a certain level, which necessarily introduces an arbitrary parameter and is costly and lengthy, or look for genes that are expressed at a constant level across all ‘normal’ tissues^1^. The use of limited numbers of tissues being examined, differences between tissues types and technological limitations means that if selected as ‘housekeeping’ genes on this basis alone they will lack specificity and selectivity.

Using the geometric mean of multiple HKG found to be stably expressed, goes some way to addressing this problem^9^. Nevertheless, there is still no consensus on which HKG is stably expressed in endometrial tissue. The reasons for the latter are firstly, that HKG expression varies with disease state, evident from different expression of HKG within the endometrium of women suffering from endometriosis, endometrial cancer and polycystic ovarian syndrome (PCOS)^1,10–12^, and secondly, the standard gene assessment used only between 6 and 12 of the most commonly cited and used HKG without due attention to appropriate normalisation within the literature. However, more recently, screened data from >30,000 microarray experiments have been made available to identify HKG that express inherent levels of stability across different tissues, using alternative experimental treatments for different disease states (geNorm Plus kit, Primerdesign)^13^. This provides a more diverse platform of genes that may be inherently stably expressed rather than relying on historical rhetoric.

The relative inefficiency of human reproduction demonstrates the potential barriers to human fertility which are as yet poorly understood^14^. As a result, there is ongoing interest into the nature of embryo-uterine signalling, factors determining the health of the embryo and the influence of uterine receptivity. Our knowledge regarding the underlying pathology behind recurrent miscarriage (RM) and recurrent implantation failure (RIF) is limited. Probing the molecular mechanism behind reproductive success will inform our understanding of the pathology and may enable the development of effective treatment. Given the caveats of previous studies in identifying HKG in endometrial tissues that is stable in both healthy and PCOS women^15^, the present study was carried out using 16 genes including the commonly used HKG as well as more recent evidence-based comparators, to analyse the stability of HKG in endometrial samples taken from women with recurrent implantation failure and recurrent miscarriage. We report for the first time HKG that are stably expressed in the endometrium in these two disease states and in those from healthy fertile individuals.

## Results

Patient demographics are shown in Table 1. There was no statistically significant difference in either age, BMI or stage of cycle of either group (RIF or RM) when compared with the controls.

**Table 1:** Patient demographics.

Demographic N = 30	Controls n = 15	RM n = 15	P value (Control vs RM)	RIF n = 15	P value (Control vs RIF)
Age (years ± S.D)	34.5 ± 5.8	37.4 ± 5.4	0.16	36.4 ± 5.3	0.35
BMI	26.6 ± 3.2	26.6 ± 6.1	0.99	26.2 ± 4.6	0.80
Stage of cycle (%)					
Follicular	6 (40)	9 (60)	0.27	5 (33)	0.71
Luteal	9 (60)	6 (40)		10 (67)	
Miscarriages (number ± S.D)	0.27 ± 4.6	3.8 ± 1.5	<0.01	NA	NA

All ten HKG demonstrated an M value below 1.5 in each of the three patient groups and there were 6 genes in the RIF group (*Ube2d2* > *Actb* > *Prdm4* > *Enox2* > *Scly* > *Gapdh*), 10 in the RM group (*Gapdh* > *Prdm4* > *Tyw*1 > *Actb* > *Scly* > *Ube4a* > *Enox2* > *Ercc6* > *Rnf20* > *Ube2d2*) and 8 in the control group (*Prdm4* > *Enox2* > *Ercc6* > *Ube2d2* > *Ube4a* > *Rnf20* > *Gapdh* > *Actb*) with an M score less than 1.0. There was no difference in the average M-value stability of the genes between the RIF (0.96 ± 0.05, n = 10) and control groups (0.81 ± 0.02, n = 10) (p = 0.08) nor RM (0.79 ± 0.07, n = 10) and controls (p = 0.89). However, if just comparing the average M-value of the top 5 most stable genes, the controls group HKG were more stable than RIF (mean difference −0.23 ± 0.05) (p < 0.01) and RM (mean difference −0.13 ± 0.04) (p < 0.01).

**Figure 1:**
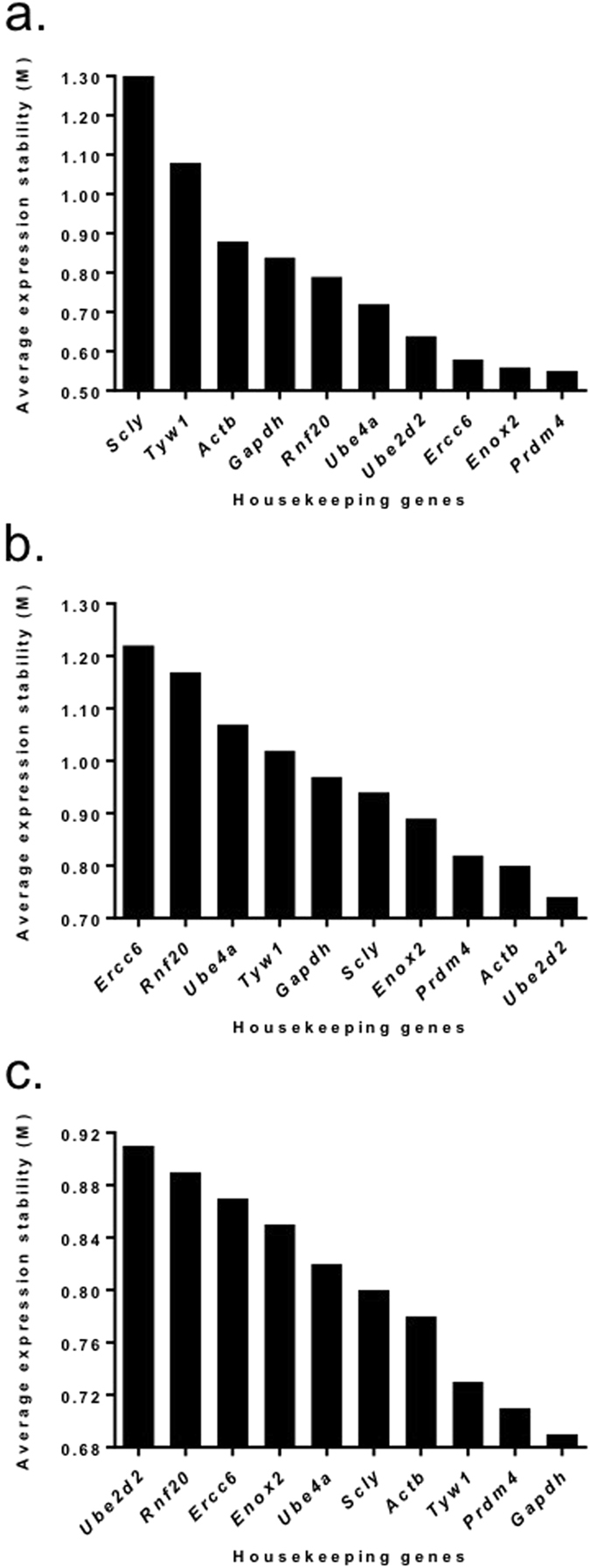
Stability of housekeeping genes (HKG) in the endometrium of individual patient groups. Graphs showing the average expression stability (M) in endometrium from (**a**) fertile women (controls), (**b**) from patients with recurrent implantation failure (RIF), and (**c**) from patients with recurrent miscarriage (RM). The HKG were ranked according to increasing stability with the most stable genes on the right lower (lowest M value). The expression stability value was calculated using the geNorm software.

**Figure 2:**
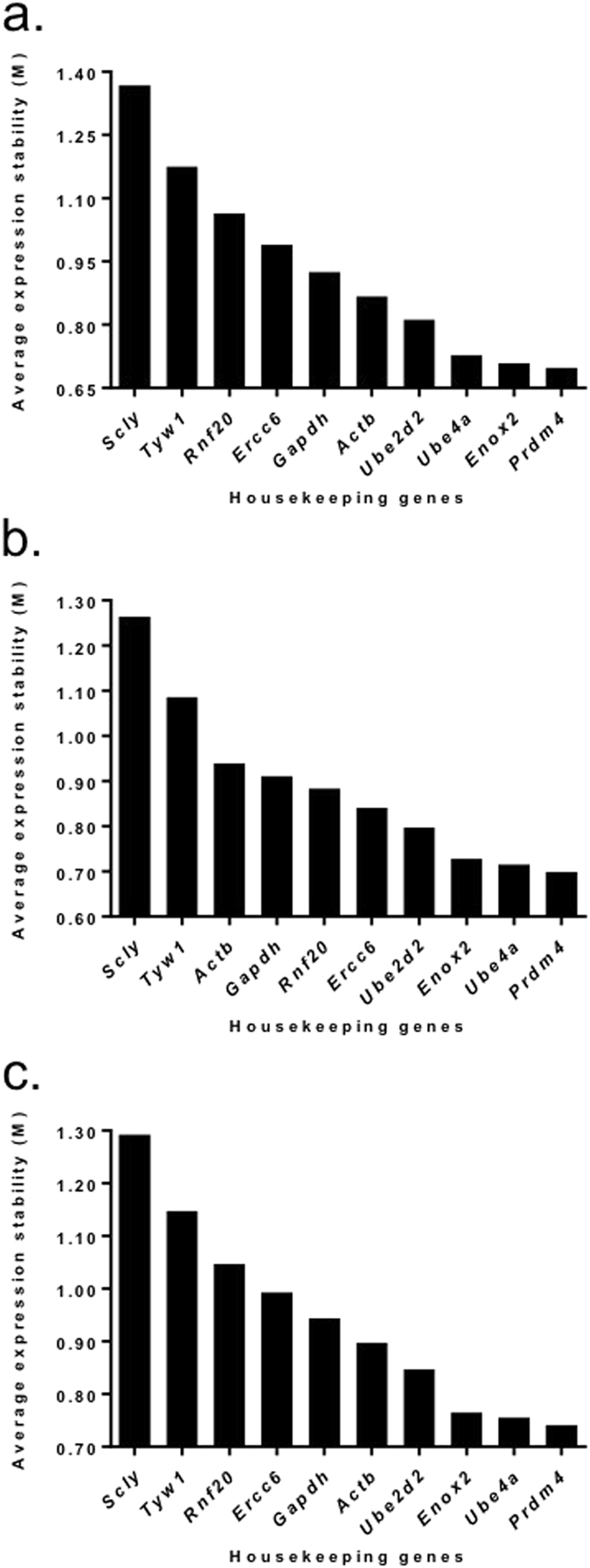
Stability of housekeeping genes (HKG) in the endometrium of combined patient groups. Graphs showing the average expression stability (M) in endometrium from (**a**) fertile women (controls) and patients with recurrent implantation failure (RIF), (**b**) fertile women (controls) and from patients with recurrent miscarriage (RM), and (**c**) fertile women (controls) and patients with recurrent implantation failure (RIF) and recurrent miscarriage (RM). The HKG were ranked according to increasing stability with the most stable genes on the right lower (lowest M value). The expression stability value was calculated using the geNorm software.

The five most stable HKG in the endometrial tissues taken from the control group were *Prdm4* > *Enox2* > *Ercc6* > *Ube2d2* > *Ube4a* (M values: 0.55, 0.56, 0.58, 0.64 and 0.72 respectively; see Fig. 1a), In the RIF women the five most stable HKG were *Ube2d* > *Actb* > *Prdm4* > *Enox2* > *Scly* (M values: 0.74, 0.80, 0.82, 0.89 and 0.94 respectively; see Fig. 1b), while in the RM women they were *Gapdh* > *Prdm4* > *Tyw*1 > *Actb* > *Scly* (M values: 0.69, 0.71, 0.73 and 0.78 and 0.80 respectively; see Fig. 1c). When taking into consideration the endometrial tissue from both control and RIF groups, the five most suitable HKG, as determined by their expression levels and minimal fluctuation, were *Prdm4* > *Enox2* > *Ube4a* > *Ube2d2* > *Actb* (M values: 0.70, 0.71, 0.73, 0.81 and 0.87, respectively; see Fig. 2a). When selecting the most suitable HKG for the control and RM groups, the five most suitable HKG were *Prdm4* > *Ube4a* > *Enox2* > *Ube2d2* > *Ercc6* (M values: 0.70, 0.72, 0.73, 0.79 and 0.84 respectively; see Fig. 2b). When combining all groups, the most stable genes were *Prdm4* > *Ube4a* > *Enox2* > *Ube2d2* > *Actb* (M values: 0.74, 0.76, 0.77, 0.85 and 0.90 respectively; see Fig. 2c).

The optimal number of HKG (reference genes) as determined by a V score below 0.15, is seen in Table 2. In this case, the lowest Vn/Vn+ 1 value is 0.15, 0.17 and 0.18 for controls, RIF and RM groups respectively (V4/5 for controls and RIF and V3/4 for RM) which means that four (for controls and RIF) or three genes (for RM) should be included according to this guideline. The addition of a sixth (for controls and RIF) or fifth (for RM) gene gave a V score below 0.15 which indicates this extra gene is unnecessary.

**Table 2:** Pairwise variation within endometrial tissue: optimal control gene number.

geNorm V	RIF	RM	Controls	RIF/Controls	RM/Controls	All groups
V2/3	0.26	0.23	0.19	0.23	0.23	0.23
V3/4	0.21	**0.18**	0.16	0.20	0.19	0.21
V4/5	**0.17**	0.14	**0.15**	**0.16**	**0.15**	**0.17**
V5/6	0.14	0.12	0.13	0.15	0.14	0.15
V6/7	0.14	0.11	0.12	0.14	0.12	0.14
V7/8	0.13	0.10	0.10	0.14	0.10	0.13
V8/9	0.12	0.09	0.19	0.16	0.17	0.15
V9/10	0.12	0.08	0.20	0.20	0.18	0.17

Pair-wise variation with the sequential addition of each housekeeping gene indicated that four genes could be used for the optimal number of reference genes in subsequent analyses in each comparison (RM compared with controls geNorm V 0.15, or RIF compared with controls geNorm V 0.16) see Table 2. A similar picture was seen when comparing all the endometrium: control, RIF and RM where the optimal number of reference targets was again four (geNorm V, 0.17) when comparing a normalization factor based on the most stable targets.

## Discussion

The present study found that 10 HKG genes not previously measured in endometrial samples were more stable than those previously identified as most stable in normal endometrium, and in the endometrium of women with RIF and RM. The most stable HKGs in the endometrium of healthy fertile women were *Prdm4* > *Enox2* > *Ercc6* > *Ube2d2* > *Ube4a*. *Ercc6* was less stable when comparing endometrium from RIF women with those from healthy fertile women, resulting in the five most stable genes becoming *Prdm4* > *Enox2* > *Ube4a* > *Ube2d2* > *Actb*. The five most stable HKG in the endometrium from RM women together with those from healthy women are *Prdm4* > *Ube4a* > *Enox2* > *Ube2d2* > *Ercc6*. When all groups were combined the most stable genes were *Prdm4* > *Ube4a* > *Enox2* > *Ube2d2* > *Actb*, and should be used as reference HKG for normalisation of endometrial tissues taken from healthy fertile patients and those with recurrent miscarriage and recurrent implantation failure. In the case of endometrial pathology other than those in RM or RIF, a wider reference HKG investigation is recommended in order to identify the best candidate HKG pertaining to that specific condition.

Previously the commonly used HKG have been applied to endometrial tissue, for example *Actb* ^16^, *1*8*s* ^17^ and *Gapdh* ^18,19^ but often studies do not describe nor comment on the suitability of the stability of the HKG in human endometrial tissue. Peptidylpropyl isomerase A (*Ppia*), Importin 8 (*Ipo8*) and Mitochondrial ribosomal protein L19 *(Mrpl19)* have been described to be the most stable genes when comparing normal and endometrial cancer cells^12^, while *Ppia* and *Gapdh* were found to be more stable HKG when comparing normal with endometriotic endometrium^20^. On the other hand, *Ywhaz, Cyc1* and *Actb* were identified as the stable HKG in the endometrium from normal and PCOS patients^11^. In the present study, the HKG *Prdm4 and Enox2 w*ere found to be the most stable HKG in the endometrium from fertile women and *Prdm4 and Enox2* in those with RIF and *Prdm4 and Ube4a* in RM. This reinforces that the expression of HKG within the endometrium is influenced by the underlying reproductive pathology. The most stable HKG overall in this study are involved in cell differentiation (*Prdm4*), protein degradation (*Ube2d2*), cell membrane transport (*Enox2*), cytoskeletal structure (*Actb*) and chromosome function (*Ube4a*) see Table 3. The level of stability of the genes has inter-group variation such that the ‘most stable’ genes in the RIF, RM and control groups are different which may reflect the heterogeneity of the diseases, again rendering normalisation a challenge.

**Table 3:** The primary function of the house keeping genes (HKG) used.

Gene name (Homosapien)	Acronym	Known Function
Actin beta*	*Actb*	Cytoskeletal structure
Glyceraldehyde- 3-phosphate dehydrogenase*	*Gapdh*	Glycolysis
Ecto-NOX disulfide-thiol exchanger 2	*Enox2*	Cell membrane transport
Excision repair cross-complementing rodent repair deficiency, comp group 6	*Ercc6*	DNA repair
PR domain containing 4	*Prdm4*	Cell differentiation
Ring Finger protein 20	*Rnf20*	Regulates chromosome structure
Selenocysteine lysase	*Scly*	Amino acid metabolism
tRNA-yW synthesizing protein 1 homolog	*Tyw1*	Ribosomal function
Ubiquitination factor E4A	*Ube4a*	Chromosome function
Ubiquitin-conjugating enzyme E2D 2	*Ube2d2*	Protein degradation

The differences in gene expression identified in this study did not vary systematically according to menstrual cycle phase, as ascribed based on reported cycle day of women with regular 28-day menstrual cycle. There is currently no consensus on the endometrial receptivity gene signature responsible for implantation success or failure^21–24^ and the variation in HKG used in different studies could account in part for these changes^25–27^. Where possible, normalising to genes with the best normalisation data to specific condition as well as cell type should be undertaken to circumvent this problem.

In the present study, the lowest Vn/Vn+ 1 values were 0.15, 0.18, 0.17 for controls, RM and RIF groups respectively, which means that five HKGs should be included for optimal use to normalise in experiments with comparisons of the tissue types described. Equally, when comparing RM and controls the lowest Vn/Vn+ 1 value was 0.15 (for V3/4) and for RIF and controls 0.16 (for V4/5) which means that four or five HKGs should be included for these experiments. However, the inclusion of more genes must also be weighed against the increasing M value and the practical limitations such as limited amounts of RNA. Furthermore, if the least-stable genes are expressed in a non-stable manner, for instance, by a variation correlated to the sample type, they will have a significant effect on the normalisation. The number of genes used for geometric averaging therefore is a trade-off between practicality and accuracy. Fewer genes, for example two, could be used but this would increase the M values to between 0.19–0.26 (compared with the threshold used of 0.15).

There is currently no consensual definition for RIF^28^ and the pathophysiology of RIF is still an unknown, although evidence shows that the embryo-endometrial interface is altered^29^. Interestingly, with respect to the order of stability of the most stable HKGs, *Ercc6* (a protein involved in DNA repair) was ranked second in RIF as opposed last in the controls. *Ercc6* is an endonuclease, part of the nucleotide excision repair (NER) pathway thought to play a role in endometrial cancer^27^. The direct link between RIF and endometrial cancer has thus far not been made^30^, although *Ercc6* is associated with an increased oxidative stress level, reactive oxidative species production and subsequent disruption of the cyclooxygenase pathway. Alteration of physiological inflammation which modulates endometrial receptivity, is a biologically plausible explanation for implantation failure^31–33^ and may be the reason that differences in the levels of Ercc6 are seen to be more stable in controls than RIF patients.

Most notable in the comparison between the stability of genes in the RM is that *Gapdh* is the most stable HKG in RM women but not in controls (seventh most stable). *Gapdh* is an enzyme involved in glycolysis and energy production and has long been used as a HKG. More constant levels of *Gapdh* in the endometrium of RM compared to controls may be an inherent aberrant energy-expensive response where there is an over-readiness of the endometrium to receive any embryo-derived signals for implantation^30,31^.

Sequencing the whole endometrial tissue is problematic. There are numerous cell types which undergo cyclic changes as a result of the postovulatory process of hormonal decidualisation. The process of endometrial remodelling occurs when progesterone exposure results in changes in all cell types that make up the endometrium, as well as an increase in endometrial leukocytes and vascular remodelling. Endometrial stromal cells differentiate from fibroblast-like cells into secretory, epithelioid and receptive decidual endometrial stromal cells in the second half of the menstrual cycle^34^. Endometrial epithelial cells can be classed as glandular or luminal epithelial cells^35^. Glandular cells undergo secretory transformation^36^ whilst luminal cells start to locally express cell adhesion receptors^37^. A difficulty of using *in-vivo* samples is the inability to interrogate each cell type individually at the molecular level. By sampling samples from the entire endometrium (as opposed to culturing and using *in-vitro* samples of one or two cell types) could arguably provide a more representative impression of the gene expression observed in human endometrium.

The timing of sampling may also be challenging as ideally, the comparison of samples should be at similar time points in each group and patient throughout the menstrual cycle. The latter poses a logistical and ethical challenge in study design. The act of biopsy may also alter gene expression of the endometrium and gene expression might vary from cycle to cycle. This problem was addressed in this study by taking endometrial biopsies from both the proliferative and secretary phases of participants. Further comparisons were performed and demonstrated no significant differences between the samples taken between the two groups and therefore acted as internal controls (see supplementary material). Whilst this study design could be criticised taking into account the above discussions, we find this study design to be the most practical, pragmatic and feasible.

In summary, we found that the most stably expressed HKG in the endometrium of healthy women and patients with RIF and RM are *Prdm4*, *Enox2*, *Ube4a* and *Ube2d2*. We therefore recommend that these HKG should be used as internal controls for qRT-PCR experiments using endometrial samples from these women in studies utilising study design similar to ours. Future studies undertaking gene expression analysis on patient cohorts with different endometrial pathologies should be aware of that certain HKG could vary in normal versus pathological tissue and suitable HKG selection strategies should be employed.

## Methods

The study was conducted under local ethical (REC number 12/SC/0548) and R&D approval (RHM O&G 0197). Patients were recruited from either elective gynecological theatre day lists or *In Vitro*-Fertilisation (IVF) clinics at Princess Anne Hospital, Southampton, UK in accordance with local guidelines. Informed consent was gained from all participants.

### Participants and Tissue Collection

Eligible participants (n = 45) were women aged between 25–45 years, attending for gynaecological procedures or fertility clinics from August 2012 to December 2013. Baseline demographics and fertility characteristics were collected for all patients. There were two independent groups of cases. The first were women who had suffered with recurrent implantation failure (women with failure of clinical pregnancy following the transfer of three or more good quality fresh or frozen embryos transferred over two or more IVF or ICSI cycles) (n = 15). The second group were women who had undergone recurrent miscarriage (women who had suffered three or more spontaneous pregnancy losses at fewer than 24 completed weeks of pregnancy) (n = 15). The third group, who served as controls, were women who were attending for elective procedures for non-endometrial pathology (n = 15) and has had at least one full term pregnancy ending in live birth without a history of recurrent implantation failure or recurrent miscarriage as previously described. Endometrial biopsies were taken by suction curette (Pipelle device, Laboratoire CCD, Paris, France), washed in sterile saline, divided into uniform size (10mm lengths), snap frozen and stored at 80 °C in separate aliquots.

#### RNA Extraction and cdna Synthesis

Total RNA was extracted from endometrial biopsies using the TRIzol (Thermo Fisher Scientific, USA). The A260/280 ratio of each sample was measured using mass spectrometry (NanoDrop; Thermo Fisher Scientific USA) and the total RNA concentration for each sample was calculated. Samples were stored at −80 °C until use. Total RNA was reverse transcribed to produce cDNA (Precision nanoScript RT kit; Primerdesign Ltd, UK) with Oligo-dT primers. The mRNA expression of the 10 genes listed in Table 3 were measured by qRT-PCR using primers and probes designed and made by Primerdesign Ltd (Southampton, UK). These sets of genes are from the geNorm Plus Reference Gene Selection kits. All samples were measured in duplicate using a LightCycler 480 Instrument (Roche Diagnostics, Germany). The optimised cycle parameters were 95 °C for 2 min, followed by 40 cycles of 95 °C for 15 seconds, 60 °C for 60 s.

### Statistical Analysis

Quantification cycle (Cq) values were transformed into relative quantification data using the delta-delta-CT method^38^. These were then converted into relative quantities (RQs) by calculating the average Cq value for replicates and using the average Cq for the given gene and the amplification efficiency. Samples for the reference gene stability analyses were divided into three groups (RIF, RM and control endometrium). To determine the stability of the HKGs a computer algorithm the geNorm Software was used (geNorm software qbasePLUS, Version: 2.6.1, Southampton UK)^2^. Part of the functionality of this program, as well as processing the transformed data and measuring gene expression is calculating gene stability. This is described as stability measure ‘M’ for a given reference gene and is generated as the average pairwise variation for that gene compared with all other tested reference genes (with a lower M value indicating more stability). Pairwise variation (V, V(n/nþ1)) determines the number of HKG required for accurate normalization and the benefit gained from additional HKG. A V score of 0.15 or below indicates that the additional gene has no significant contribution to the newly calculated normalization factor and is therefore not needed^2,9^. M values were further analysed using the Prism Software (Version 7.0b, GraphPad Software, Inc). Group differences were evaluated using independent samples t-test to elucidate differences in both patient groups and differences in M-Values. P < 0.05 was considered statistically significant. Data values are represented as mean ± standard deviation (SD).

## Electronic supplementary material

Supplementary Information
