# Supplementary material for: Identifying stably expressed housekeeping genes in the endometrium of fertile women, women with recurrent implantation failure and recurrent miscarriages
Source: Sci Rep. 2017 Nov 1;7:14857. doi: 10.1038/s41598-017-07901-6 (PMC5665911; doi:10.1038/s41598-017-07901-6)
Supplement: Supplementary file 1 — Supplementary Information [file 41598_2017_7901_MOESM1_ESM.docx]

Supplementary data for: ‘Identifying stably expressed housekeeping genes in the endometrium of fertile women, women with recurrent implantation failure and recurrent miscarriages’, Stocker et al.

Table A

A comparison of the number of women sampled in the two halves of the menstrual cycle. This demonstrates there was no difference in the distribution of the women.

| **Stage of cycle** |  |  |  |
| --- | --- | --- | --- |
| Group | Stage of cycle | Frequency | Percentage |
| Control | Proliferative | 6 | 40.0 |
|  | Secretory | 9 | 60.0 |
|  | Total | 15 | 100.0 |
| RM | Proliferative | 9 | 60.0 |
|  | Secretory | 6 | 40.0 |
|  | Total | 15 | 100.0 |
| RIF | Proliferative | 5 | 33.3 |
|  | Secretory | 10 | 66.7 |
|  | Total | 15 | 100.0 |

Difference in numbers in proliferative vs secretory groups:

1. between controls and RM P=0.29
2. between controls and RIF P=0.72

Table B

A comparison between the numbers sampled between the two weeks in the secretary cycle. They do not vary enough for us to consider grouping them day by day.

| **Secretory Phase** |  |  |  |
| --- | --- | --- | --- |
|  |  | Frequency | Percentage |
|  | Early secretory (D14-21) | 12 | 48.0 |
|  | Late secretory (D22-0) | 13 | 52.0 |
|  | Total | 25 | 100.0 |

Difference in numbers in early vs late secretory groups:

1. between controls and RM P=0.27
2. between controls and RIF no difference
